# *CYP2B6* allelic variants and non-genetic factors influence CYP2B6 enzyme function

**DOI:** 10.1038/s41598-022-07022-9

**Published:** 2022-02-22

**Authors:** Katalin Mangó, Ádám Ferenc Kiss, Ferenc Fekete, Réka Erdős, Katalin Monostory

**Affiliations:** 1 Institute of Enzymology, Research Centre for Natural Sciences, Magyar Tudósok 2, Budapest, 1117 Hungary; 2 Doctoral School of Pharmaceutical Sciences, Semmelweis University, Budapest, Hungary

**Keywords:** Medical research, Molecular medicine

## Abstract

Human CYP2B6 enzyme although constitutes relatively low proportion (1–4%) of hepatic cytochrome P450 content, it is the major catalyst of metabolism of several clinically important drugs (efavirenz, cyclophosphamide, bupropion, methadone). High interindividual variability in CYP2B6 function, contributing to impaired drug-response and/or adverse reactions, is partly elucidated by genetic polymorphisms, whereas non-genetic factors can significantly modify the CYP2B6 phenotype. The influence of genetic and phenoconverting non-genetic factors on CYP2B6-selective activity and CYP2B6 expression was investigated in liver tissues from Caucasian subjects (N = 119). Strong association was observed between hepatic *S*-mephenytoin *N*-demethylase activity and CYP2B6 mRNA expression (*P* < 0.0001). In less than one third of the tissue donors, the CYP2B6 phenotype characterized by *S*-mephenytoin *N*-demethylase activity and/or CYP2B6 expression was concordant with *CYP2B6* genotype, whereas in more than 35% of the subjects, an altered CYP2B6 phenotype was attributed to phenoconverting non-genetic factors (to CYP2B6-specific inhibitors and inducers, non-specific amoxicillin + clavulanic acid treatment and chronic alcohol consumption, but not to the gender). Furthermore, CYP2B6 genotype–phenotype mismatch still existed in one third of tissue donors. In conclusion, identifying potential sources of CYP2B6 variability and considering both genetic variations and non-genetic factors is a pressing requirement for appropriate elucidation of CYP2B6 genotype–phenotype mismatch.

## Introduction

The function of cytochrome P450 (CYP) enzymes, one of the major catalysts in drug metabolism, is significantly influenced by genetic polymorphisms leading to substantial inter-individual variability in drug response and/or adverse reactions. Although human CYP2B6 constitutes only 1–4% of hepatic CYP protein content, it is responsible for the metabolism of some clinically important drugs, including the antidepressant bupropion, the antiretroviral efavirenz, the anticancer cyclophosphamide, the analgesic ketamine and methadone^1–4^. *N*-Demethylation of the anticonvulsant *S*-mephenytoin to nirvanol and hydroxylation of bupropion as well as the oxidative hydroxylation of efavirenz are used as probe substrates and selective reactions for in vitro characterization of CYP2B6 activity^5–7^. Catalytic activity and expression of CYP2B6 are highly variable between individuals that have been reported to be primarily impacted by genetic polymorphisms of *CYP2B6*. The PharmVar (Pharmacogene Variation Consortium) website lists 38 alleles, several of which are associated with increased, reduced or no enzyme activity (https://www.pharmvar.org/gene/CYP2B6, access date: 24.01.2022). Moreover, significant interethnic differences in the frequencies of clinically most relevant *CYP2B6* alleles have been demonstrated^8^ (https://www.pharmgkb.org/page/cyp2b6RefMaterials, access date: 24.01.2022). PharmVar has published information about *CYP2B6* allele functionality and *CYP2B6* diplotype to phenotype estimation^9^. A standardized and easily interpreted classification of the phenotypic characteristics for each *CYP2B6* allelic variant has also been provided. CYP2B6*22 variant contains a promoter mutation (rs34223104, g.-82 T > C) leading to an enhanced expression of *CYP2B6* gene through an altered transcription mechanism^10^. Zukunft et al. ^11^ reported increased CYP2B6 transcription and bupropion hydroxylase activity in liver samples of CYP2B6*1/*22 heterozygous subjects. CYP2B6*4 allele contains the Lys262Arg change (rs2279343, g.18053A > G) that results in a structurally altered CYP2B6 enzyme variant and an increase in CYP2B6 enzyme activity (efavirenz hydroxylation)^9,12–14^. The in vivo clearance of methadone, bupropion and efavirenz was also demonstrated to be increased in individuals carrying CYP2B6*4^15–17^. It should be noted that CYP2B6*4 allele displayed decreased metabolic activity toward cyclophosphamide comparing to CYP2B6*1^18^. CYP2B6*6 allele is defined by two single nucleotide variations (SNVs). In CYP2B6*6, the same gain-of-function Lys262Arg amino acid substitution (rs2279343, g.18053A > G) was identified as in CYP2B6*4. In addition, the g.15631G > T change (rs3745274, Gln172His) in CYP2B6*6 alters the normal mRNA splicing process that decreases the hepatic expression of CYP2B6 mRNA and enzyme protein, and consequently CYP2B6 activity^19^. The effect of g.18053A > G change in CYP2B6*4 allele seems to be reversed by the g.15631G > T in CYP2B6*6^18^. Decreased clearance of *S*-methadone and efavirenz was reported in the presence of one or two CYP2B6*6 alleles, while reduced hydroxylation of bupropion enantiomers was observed only in CYP2B6*6/*6 carriers^15–17^. The g.15631G > T (rs3745274, Gln172His) nucleotide substitution was detected in CYP2B6*9 allele resulting in decreased enzyme activities (both bupropion and efavirenz hydroxylation) similarly to CYP2B6*6^20^; however, information about the clinical significance of CYP2B6*9 is hardly available because of its low prevalence in all populations. The g.25505C > T (rs3211371, Arg487Cys) nucleotide change in CYP2B6*5 allele-variant has been reported to display mild or negligible effect on CYP2B6 catalytic activity, although it seemed to influence the CYP2B6 protein expression^15–17,21,22^.

The well-defined allele-functionality definition criteria promote the designation of clinically adaptable dosing recommendations for CYP2B6 substrates^9^. Clinical Pharmacogenetics Implementation Consortium (CPIC) has published the therapeutic recommendation guideline for efavirenz based on *CYP2B6* genotype distinguishing ’poor’, ‘intermediate’, ’normal’, ’rapid/ultra-rapid’ CYP2B6 metabolizer phenotypes^23^. The genetically determined CYP2B6 expression and activities however are often modified by CYP2B6 specific or non-specific non-genetic factors. Co-medication for example can decrease or even increase CYP function; therefore, phenoconversion of CYP genes should be taken into account during prediction of drug-metabolizing capacity^2,24^. Thiotepa, ticlopidine, clopidogrel and sertraline are well-known CYP2B6 inhibitors resulting in substantial reduction of CYP2B6 activities, whereas phenobarbital, rifampicin, phenytoin, dexamethasone and several corticosteroids can activate the nuclear receptors CAR (constitutive androstane receptor) and PXR (pregnane X receptor) upregulating CYP2B6 transcription and increasing CYP2B6 enzyme activity^25–29^. Furthermore, several studies suggested that gender is an intrinsic factor which may have a considerable impact on CYP2B6 expression and/or activity, while others did not confirm the role of gender in CYP2B6 function^19,30–34^. Medication with CYP2B6-selective inhibitors or inducers is one of the major causes of phenoconversion; however, certain pathological conditions and co-morbidities can also contribute to phenoconversion of drug-metabolizing enzymes^35–37^. Various liver diseases (alcohol-related or non-alcoholic liver diseases), cancer and other inflammatory conditions have been reported to impact the patients’ drug-metabolizing capacity^38,39^. For appropriate estimation of CYP2B6-mediated drug metabolism, the evaluation of both genetic (CYP2B6 SNVs/haplotypes) and non-genetic factors (CYP2B6-selective inhibitor or inducer therapy; non-specific factors: sex, morbidities, amoxicillin + clavulanic acid therapy) is required. The main aim of the present study was to investigate the potential impact of *CYP2B6* allelic variants most common in Caucasian populations on CYP2B6 activity and mRNA expression in human liver tissues. The hepatic microsomal activity was characterized by the CYP2B6-selective *S*-mephenytoin *N*-demethylation. Furthermore, we attempted to identify non-genetic factors including demographic parameters and co-medication that can modify CYP2B6 phenotype predicted from genotype.

## Materials and methods

### Human liver samples

Human liver tissues (N = 119) were obtained from donated organs at the Department of Transplantation and Surgery, Semmelweis University (Budapest, Hungary). The study was approved by the Hungarian Committee of Science and Research Ethics, Medical Research Council (125/PI/2011, 4799-0/2011EKU), and was performed in accordance with the relevant guidelines and regulations (Act CLIV of 1997 on Health, decree 23/2002 of the Minister of Health of Hungary and the declaration of Helsinki). The subjects' demographic and clinical data (sex, age, cause of death, acute and chronic medication prior the explantation, smoking and alcohol consumption status) were recorded (Supplementary Table 1). Liver tissues from those subjects who were recorded chronic alcohol consumption [N = 11] were evaluated to be fibrotic due to alcohol related liver disease. Human livers were perfused with Euro-Collin’s solution (Fresenius SE & Co. KGaA, Bad Homburg vdH, Germany) and excised. The tissues (approximately 1 g) were homogenized in 0.1 mM Tris–HCl buffer (pH 7.4) containing 1 mM EDTA and 154 mM KCl. Microsomal fraction was isolated by differential centrifugation and protein content of microsomes was determined by the method of Lowry et al. using bovine serum albumin as the standard^40,41^. Approximately 50 mg of liver tissue were homogenized in TRIzol reagent (Thermo Fisher Scientific, Waltham, MA) and total RNA was isolated according to the manufacturer’s instructions. The hepatic RNA samples were stored in ultra-pure water containing 0.1% diethylpyrocarbonate at − 80 °C for further analyses.

### CYP2B6 enzyme activity assay

The *S*-mephenytoin *N*-demethylation activity selective for CYP2B6 were performed in the incubation mixture containing NADPH-generating system (1 mM NADPH, 10 mM glucose-6-phosphate, 5 mM MgCl_2_ and 2 units/ml glucose-6-phosphate dehydrogenase), human liver microsomes (0.8 mg/ml protein) and *S*-mephenytoin (1 mM). After 40-min incubation at 37 °C, the enzyme reactions were terminated by ice-cold acetonitrile, and the incubation mixtures were centrifuged at 10.000×*g* for 10 min. Formation of nirvanol was quantified by high-performance liquid chromatography according to Heyn et al.^5^. CYP2B6 enzyme assay for each donor was performed in triplicate, and the activity was expressed as pmol nirvanol*(mg protein)^-1^*min^-1^.

### CYP2B6 genotyping

Genomic DNA templates were isolated from liver tissues using Quick-DNA Miniprep Plus Kit (Zymo Research, Irvine, CA). The following *CYP2B6* polymorphisms were determined using validated TaqMan™ Drug Metabolism Genotyping Assays (Thermo Fisher Scientific) for g.-82 T > C (rs34223104, C_27830964_10), g.15631G > T (rs3745274, C_7817765_60) and g.25505C > T (rs3211371, C_30634242_40). Each reaction (in 5-µl reaction volume) contained Luminaris Probe qPCR Master Mix (Thermo Fisher Scientific), TaqMan™ Drug Metabolism Genotyping Assay (Thermo Fisher Scientific), 10–15 ng genomic DNA sample and nuclease-free water and incubated at 50 °C for 2 min and 95 °C for 10 min; and in 50 cycles of 95 °C for 15 s and 60 °C for 1 min. For identification of g.18053A > G nucleotide substitution (rs2279343), no validated PCR (polymerase chain reaction) primers and probes are commercially available; therefore, a two-step PCR assay based on the ‘nested’ PCR method with ‘touchdown’ thermal cycling protocol and the TaqMan PCR was developed (Supplementary Fig. 1A)^42,43^. In the PCR reactions, two sets of primer pairs were applied and used consecutively to increase the *CYP2B6* specificity of the SNV-discrimination and to avoid the amplification of *CYP2B7P* (Table 1). The first step was a ’nested’ PCR containing PCRBIO VeriFi Master Mix (PCR Biosystems Ltd., London, UK), 400–400 nM forward and reverse primers (first set of primer pairs, Table 1) and 40–50 ng genomic DNA template. The first primer pair generated a relatively long (1275-bp) amplicon containing the whole *CYP2B6* exon 5 and exon 6 with the intron 5 in between and with some short surrounding upstream and downstream intron region sequences (Supplementary Fig. 1A). The homology of *CYP2B6* intron sequences with *CYP2B7P* is somewhat lower than that of the exons^44^; therefore, the first primer pair designed for the upstream and downstream intron regions (in introns 4 and 6) with the ‘touchdown’ PCR thermal cycling protocol was expected to provide the *CYP2B6* gene specific hybridization of the primers. The thermal cycling protocol was performed according to the principles of ‘touchdown’ PCR: 95 °C for 1 min and 10 cycles of 95 °C for 15 s, 72–62 °C for 15 s (decreasing 1 °C/cycle), 72 °C for 1 min, and 10 cycles of 95 °C for 15 s, 62 °C for 15 s, 72 °C for 1 min. During the initial cycle, an annealing temperature (72 °C) higher than the targeted melting temperature of primers (62 °C) was used. Afterwards, the annealing temperature was decreased progressively over 10 cycles which made the reaction conditions more permissive. Theoretically, each cycle with decreasing annealing temperature by 1 °C produced four-fold exponential differences between correct and incorrect annealing resulting in the enrichment of the *CYP2B6* specific over the non-target *CYP2B7P* specific product^42^. For testing *CYP2B6* specificity of the ‘nested’ PCR reaction, the three verifying primers designed by Jacob et al. were modified: (1) a *CYP2B6* specific (t5b6_2) forward primer, (2) a *CYP2B7P* specific (t5b7_2) forward primer and (3) a common (t5con_2) reverse primer (Table 1)^45^. The optimization of the length, GC-content and the melting temperatures of these verifying primers provided more suitable reaction conditions than those published by Jacob et al.^45^. Using genomic DNA or DNA amplicon produced in the ‘nested’ PCR as the template, great differences in Ct (threshold cycle) values were observed between *CYP2B6* and *CYP2B7P* specific products (Ct for genomic DNA: 23.1 and 24.1 vs. Ct for DNA amplicon: 23.1 and 37.04) (Supplementary Fig. 1B). It confirmed that substantial amount of *CYP2B6* specific amplicon (more than 5000-fold) was produced in the ‘nested’ PCR comparing to *CYP2B7P* specific amplicon, whereas for *CYP2B6-*selective amplification, the genomic DNA appeared to be not an appropriate template. The second PCR was carried out using Luminaris Probe qPCR Master Mix (Thermo Fisher Scientific), 300–300 nM forward and reverse primers (second set of primer pairs), 200–200 nM ’wild’ and ’mutant’ TaqMan probes (Table 1) and the 100-fold dilution of the ‘nested’ PCR product as the template. The incubation protocol was 50 °C for 2 min, 95 °C for 10 min and 50 cycles of 95 °C for 15 s, 66 °C for 1 min. The second primer pair generating a short, 137-bp amplicon with the rs2279343 mutation site in exon 5 and TaqMan probes designed for the wild-type and the mutant type of rs2279343 were used for SNV-discrimination (Supplementary Fig. 1C). The in silico design and validation of the primers and probes (Table 1) were performed by IDT Oligoanalyzer (https://eu.idtdna.com/calc/analyzer, access date: 24.01.2022), NCBI Primer-BLAST (https://www.ncbi.nlm.nih.gov/tools/primer-blast/, access date: 24.01.2022) and NCBI Nucleotide BLAST (https://blast.ncbi.nlm.nih.gov/Blast.cgi, access date: 24.01.2022) software tools. The oligonucleotides were synthesized by Eurofins Genomics Germany GmbH (Ebersberg, Germany). The length of the amplicons in each reaction was verified using the TapeStation 4200 instrument (Agilent Technologies Inc., Santa Clara, CA). The *CYP2B6* specificity of the first ‘nested’ PCR step was confirmed by using *CYP2B6* and *CYP2B7P* gene specific forward primers and a common reverse primer (Table 1) with SYBR Green detection. The accuracy of SNV-discrimination of homozygous wild-type (g.18053A/A), heterozygous (g.18053A/G) and homozygous mutant type (g.18053G/G) samples was confirmed by Sanger-sequencing (Eurofins Genomics Germany GmbH) (Supplementary Fig. 2).

**Table 1:** Oligonucleotide sequences for the *CYP2B6* rs2279343 SNV analysis and CYP2B6 mRNA expression quantification.

*CYP2B6* rs2279343 analysis	Oligonucleotides	Sequences (5'- > 3')	Length of amplicons^a^
Step 1: ‘nested’ PCR	Fw primer	ACA GGC TGA GGT AGA CAA TG	1275 bps
	Rev primer	CTC AGA AGG AGG TCA GAA GAC	
Step 2: TaqMan PCR	Fw primer	GGC ACA CAG GCA AGT TTA CA	137 bps
	Rev primer	CTT TTT CCA TGT GGA GCA GGT AG	
	W probe	FAM-CGC CCC CAA GGA CCT CAT CGA CA-BHQ1	
	M probe	HEX-CGC CCC CAG GGA CCT CAT CGA-BHQ1	
Verifying primers for *CYP2B6* specificity^b^	t5b6_2 Fw primer	**AG**T TAG AGA TAC GCG GTT GGA TG	294 bps
	t5b7_2 Fw primer	TTA GAG ATG TGC AGC TGG ACA **T**	292 bps
	t5con_2 Rev primer	**TAT TTG** AGC ATG AGC AGG AAG C	
**mRNA expression**			
CYP2B6	Fw primer	AAA GCG GAG TGT GGA GGA	93 bps
	Rev primer	AAG GTG GGG TCC ATG AGG	
	Probe	FAM-AGG AGG AG-BHQ1	
GAPDH (reference)	Fw primer	AGC CAC ATC GCT CAG ACA C	66 bps
	Rev primer	GCC CAA TAC GAC CAA ATC C	
	Probe	HEX-TGG GGA AGG TGA AGG TCG-BHQ1	

*Fw* forward, Rev reverse, *W* wild, *M* mutant, FAM, HEX fluorophores, BHQ1 quencher.

^a^Length of amplicons were determined by NCBI Primer Blast.

^b^Sequences of verifying primers based on Jacob et al.^45^ with slight modifications (in bold).

### Analysis of CYP2B6 mRNA expression by quantitative real-time PCR

Hepatic RNA samples (3 µg) were reverse transcribed into cDNA using iScript cDNA synthesize kit (Bio-Rad Laboratories Inc., Hercules, CA). The real-time PCR was performed by KAPA Probe Fast qPCR Master Kit™ (Merck KGaA, Darmstadt, Germany) and UPL probes for CYP2B6 (Roche Diagnostics GmbH, Mannheim, Germany). The SNV g.15631G > T (rs3745274) is associated with an aberrant mRNA splicing variant lacking exons 4 to 6 and entailing reduced CYP2B6 function^19^. Our aim was to detect only the full-length CYP2B6 mRNA and to distinguish it from the aberrant splicing variant; therefore, the primer pair for CYP2B6 expression assay was designed to the exons 3 and 4 (forward and reverse primers, respectively), and the intron-spanning amplicon detected exclusively the functional full-length mRNA variant (Table 1). The quantity of the target mRNA relative to that of the housekeeping gene glyceraldehyde 3-phosphate dehydrogenase (GAPDH) was determined. The sequences of primers and probes used for the real-time PCR analyses of CYP2B6 and GAPDH expression are shown in Table 1.

### Data analysis

The frequency distribution of *S*-mephenytoin *N*-demethylase activity was determined in liver tissue samples, and three categories (low, intermediate and high) were distinguished for poor, intermediate and extensive metabolizers (PM, IM and EM). Liver tissue samples were screened for *CYP2B6* polymorphisms (CYP2B6*4, CYP2B6*5, CYP2B6*6, CYP2B6*9 and CYP2B6*22). *CYP2B6* allele discrimination was performed by Bio-Rad CFX Maestro Software 1.1 (v4.1.2433.1219; Bio-Rad Laboratories), and *CYP2B6* haplotypes were estimated by PHASE software v2.1.1.^46,47^. To improve the accuracy of haplotype estimation, the *CYP2B6* genotype data of 44 patients studied by Dobrinas et al. were used in addition to the data obtained in the present study^48^. InStat v3.06 (GraphPad Software, Inc., San Diego, CA) was used for analysing the associations between CYP2B6 activity and gender, *CYP2B6* genotypes and CYP2B6 activity or mRNA expression as well as CYP2B6 mRNA expression and activity using Kruskal–Wallis ANOVA followed by Dunn’s multiple comparisons test. Phenoconversion frequencies were evaluated on the basis of the frequencies of the non-genetic factors (medication, chronic alcohol consumption) in the medical histories of tissue donors in various genotype-based phenotype groups by Fisher’s exact test. Linear regression models were formulated to test potential associations between CYP2B6 activity (N = 105) or mRNA expression (N = 93) as dependent variables and *CYP2B6* SNVs, haplotypes, sex, chronic alcohol consumption and medications (CYP2B6 inducer therapy, amoxicillin + clavulanic acid therapy) as co-variates. Multiple linear regression analyses were carried out by IBM SPSS Statistics software [v28.0.1.0 (142), IBM Corp., Armonk, NY]. *P* value < 0.05 was considered to be statistically significant.

## Results

### Genetic variability of CYP2B6

Liver tissues (N = 119) were screened for the g.-82 T > C (rs34223104), g.15631G > T (rs3745274), g.18053A > G (rs2279343) and g.25505C > T (rs3211371) SNVs of *CYP2B6* using real-time PCR analyses, and CYP2B6 haplotypes were identified for CYP2B6*4, CYP2B6*5, CYP2B6*6, CYP2B6*9 and CYP2B6*22. A novel method based on ‘nested’ PCR using ‘touchdown’ PCR thermal protocol and SNV-discrimination using TaqMan probes was developed for identification of g.18053A > G^42,43^. The wild-type CYP2B6*1 allele was assigned to g.-82 T/15631G/18053A/25505C haplotype. The most prevalent alleles were CYP2B6*6 and CYP2B6*5, whereas CYP2B6*4, CYP2B6*9 and CYP2B6*22 occurred with much lower frequencies (Table 2). According to the phenotype prediction by PharmVar (Table 3), half of the tissue donors carrying CYP2B6*1/*1 or CYP2B6*1/*5 genotypes (38.7% or 12.6%) (Table 2) were considered to be ’normal’ metabolizers. More than one third (36.1%) of the subjects with one normal and one decreased function alleles (CYP2B6*1/*6, CYP2B6*5/*6, CYP2B6*1/*9 and CYP2B6*4/*6 genotypes) were ’intermediate’ metabolizers. Those donors with two loss-of-function alleles (CYP2B6*6/*6 and CYP2B6*6/*9 genotypes) were referred to be ’poor’ metabolizers (7.5%), whereas an additional group carrying one normal and one gain-of-function alleles (CYP2B6*1/*4, CYP2B6*1/*22 and CYP2B6*4/*5 genotypes) were distinguished to be ’rapid/extensive’ metabolizers (5%) (Tables 2 and 3).

**Table 2:** *CYP2B6* allele and genotype frequencies in liver tissue donors and in Caucasian populations.

	N	Frequency (%)	
		Tissue donors	Caucasian populations^a^
***CYP2B6* alleles**			
*4	5	2.1	2.2–6.2
*5	25	10.5	9–12.2
*6	59	24.8	7–28.15
*9	2	0.8	0–1.47
*22	2	0.8	1.4–2.4
***CYP2B6* genotypes**			
*1/*1	46	38.7	21.05–24.1
*1/*6	32	26.9	22.1–22.9
*1/*5	15	12.6	7.36–11.3
*5/*6	9	7.6	5.3–7.36
*6/*6	8	6.7	5.4–7.36
*1/*4	3	2.5	1.05–4
*1/*22	2	1.7	1.3–2.1
*1/*9	1	0.8	< 1.4
*6/*9	1	0.8	< 0.8
*4/*5	1	0.8	< 0.9
*4/*6	1	0.8	< 2.1

^a^Based on PharmVar (https://www.pharmgkb.org/page/cyp2b6RefMaterials)^8^.

**Table 3:** Genotype-based prediction of CYP2B6 metabolizer phenotypes according to PharmVar and phenoconversion by non-genetic factors.

*CYP2B6* genotype	CYP2B6 phenotype		Phenoconversion		
	*CYP2B6* genotype-based prediction^a^	CYP2B6 activity categories	medication with CYP2B6 inducer^b^	medication with CYP2B6 inhibitor^c^	non-specific non-genetic factors^d^
*6/*6 *6/*9	Poor	PM	IM-EM	PM	PM
*1/*6 *5/*6 *1/*9 *4/*6	Intermediate	low IM	high IM-EM	PM	PM
*1/*1 *1/*5	Normal	high IM	EM	low IM-PM	low IM-PM
*1/*4 *1/*22 *4/*5	Rapid/ Ultrarapid	EM	EM	high IM	high IM

*PM* poor metabolizer, *IM* intermediate metabolizer, *EM* extensive metabolizer.

^a^According to the CPIC.

^b^CYP2B6 inducers: dexamethasone, methylprednisolone, prednisolone, hydrocortisone, cortisone, midazolam, felodipine, diazepam.

^c^CYP2B6 inhibitor: amlodipine.

^d^Non-specific factors: chronic alcohol consumption, amoxicillin + clavulanic acid therapy.

### Hepatic CYP2B6 activity and mRNA expression

**Figure 1:**
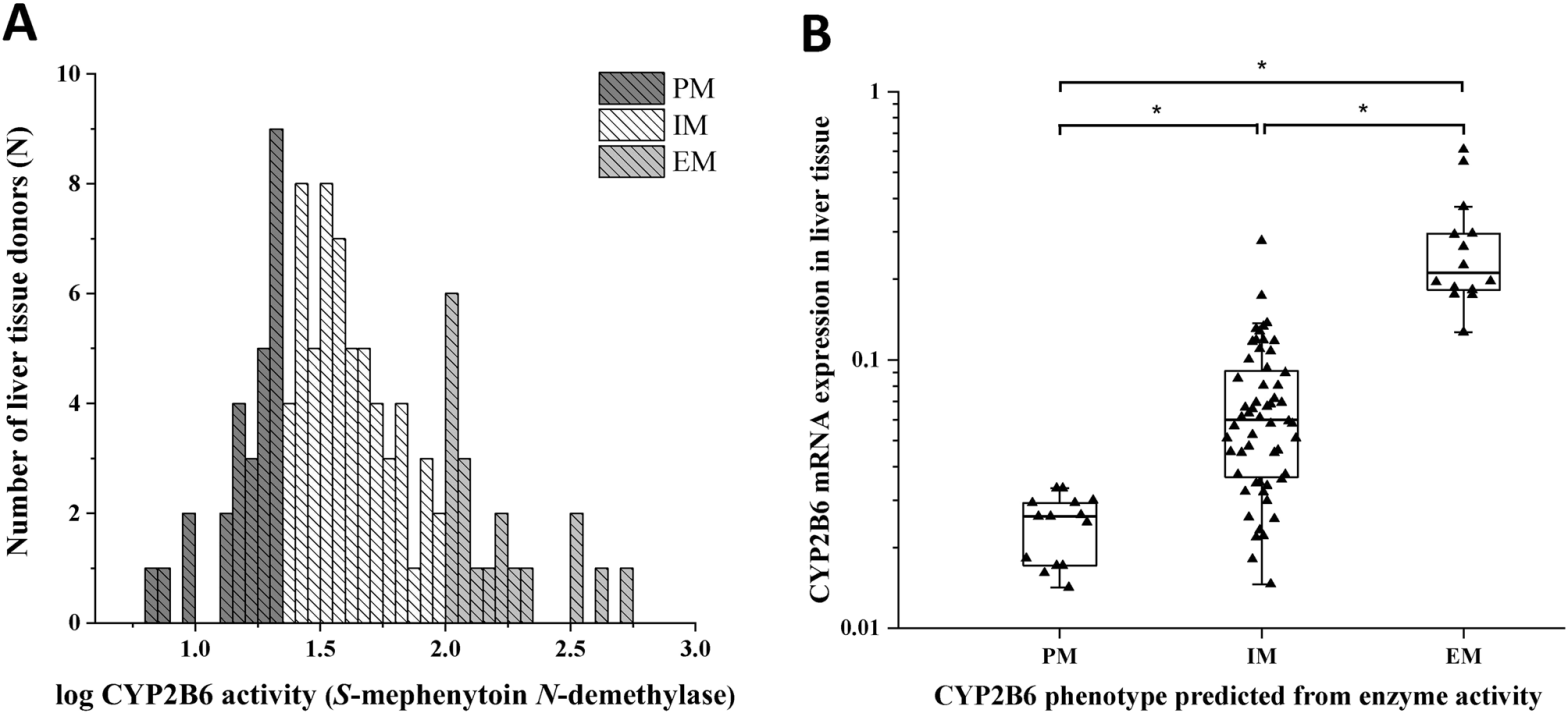
Frequency distribution of hepatic CYP2B6 activities (*S*-mephenytoin *N*-demethylation) (N = 105) (**A**) and association between CYP2B6 activities and mRNA expression (N = 85) (**B**) in human tissue donors. PM poor metabolizer; IM intermediate metabolizer; EM extensive metabolizer. * Significant difference (*P* < 0.0001).

**Figure 2:**
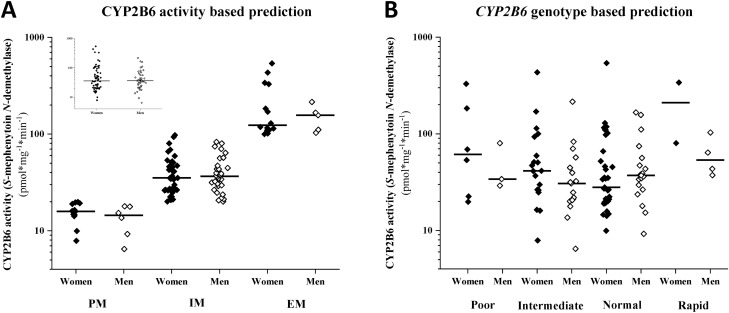
Gender-based differences in *S*-mephenytoin *N*-demethylation between various CYP2B6 metabolizer groups (**A**) and between *CYP2B6* genotype groups (**B**). The inserted graph (**A**) displays the differences in CYP2B6 activities between men and women.

Hepatic microsomal *S*-mephenytoin *N*-demethylase activity selective for CYP2B6 was determined in 105 tissue donors. CYP2B6 activities varied from 6.47 to 538.3 pmol*mg^-1^*min^−1^, representing approximately 85-fold variability and showed skewed distribution (Fig. 1A). Low, intermediate and high activities were distinguished using the cut-off values of 20 pmol*mg^−1^*min^−1^ and 99.9 pmol*mg^−1^*min^−1^ for poor, intermediate and extensive metabolizers. Approximately two thirds of the liver tissues (65%) displayed intermediate *S*-mephenytoin *N*-demethylase activity, whereas of 105 tissue donors, 18 and 19 showed low and extensive CYP2B6 activities, respectively. Furthermore, significant differences in hepatic CYP2B6 mRNA expression were observed between various CYP2B6 activity groups (N = 84, *P* < 0.0001) (Fig. 1B). Demographic parameters, such as age (data not shown) or sex (Fig. 2, Table 4), appeared not to influence CYP2B6 activity. No significant differences between men and women were observed in *S*-mephenytoin *N*-demethylase activity, and the distribution of men and women in the activity categories or in genotype-based categories were found to be homogenous (Fig. 2A,B).

**Table 4:** Multivariate analysis on CYP2B6 activity (*S*-mephenytoin *N*-demethylation) considering genetic (*CYP2B6* SNVs or haplotypes) and non-genetic factors.

Variable	CYP2B6 activity			CYP2B6 mRNA expression		
	Coefficient B (SE)	Coefficient ß	*P* value	Coefficient B (SE)	Coefficient ß	*P* value
**SNVs, non-genetic**						
Constant	67.09 (13.86)		< 0.001	0.12 (0.02)		< 0.001
g.-82 T > C (rs34223104)	32.10 (56.79)	0.054	0.57	0.02 (0.07)	0.021	0.82
g.15631G > T (rs3745274)	− 79.98 (38.01)	− 0.485	**0.03**	− 0.16 (0.05)	− 0.728	**0.004**
g.18053A > G (rs2279343)	63.33 (36.59)	0.386	0.08	0.11 (0.05)	0.496	**0.04**
g.25505C > T (rs3211371)	8.69 (18.76)	0.044	0.64	− 0.04 (0.03)	− 0.124	0.19
Sex	− 15.34 (16.01)	− 0.092	0.34	− 0.01 (0.02)	− 0.052	0.59
Alcohol consumption	− 33.12 (26.75)	− 0.119	0.21	− 0.06 (0.03)	− 0.174	0.07
Amoxicillin/clavulanic acid therapy	− 35.03 (28.38)	− 0.120	0.22	− 0.06 (0.04)	− 0.148	0.14
Inducer therapy	65.90 (22.73)	0.311	**0.005**	0.11 (0.03)	0.391	** < 0.001**
**Haplotype, non-genetic 1**						
Constant	67.09 (13.89)		< 0.001	0.12 (0.02)		< 0.001
g.-82 T/15631G/**18053G**/25505C	73.03 (41.42)	0.171	0.08	0.09 (0.05)	0.172	0.08
g.-82 T/15631G/18053A/**25505 T**	8.81 (18.84)	0.045	0.64	− 0.03 (0.03)	− 0.122	0.21
g.-82 T/**15631 T**/**18053G**/25505 T	− 15.60 (17.82)	− 0.094	0.38	− 0.05 (0.02)	− 0.245	**0.02**
g.-82 T/**15631 T**/18053A/25505C	− 47.28 (59.30)	− 0.079	0.43	− 0.08 (0.07)	− 0.111	0.27
g.-**82C**/15631G/18053A/25505C	33.12 (56.92)	0.055	0.56	0.02 (0.07)	0.022	0.82
Sex	− 17.38 (16.16)	− 0.105	0.29	− 0.02 (0.02)	− 0.077	0.44
Alcohol consumption	− 33.37 (26.81)	− 0.120	0.21	− 0.06 (0.03)	− 0.164	0.09
Amoxicillin/clavulanic acid therapy	− 34.20 (28.48)	− 0.117	0.22	− 0.06 (0.04)	− 0.148	0.14
Inducer therapy	66.31 (23.41)	0.313	**0.006**	0.11 (0.03)	0.398	** < 0.001**
**Haplotype, non-genetic 2**						
Constant	67.30 (13.70)		< 0.001	0.12 (0.02)		< 0.001
g.-82 T/15631G/**18053G**/25505C	74.52 (40.75)	0.175	0.07	0.09 (0.05)	0.177	0.07
g.-82 T/15631G/18053A/**25505 T**	9.34 (18.65)	0.047	0.62	− 0.03 (0.03)	− 0.117	0.22
g.-82 T/**15631 T**/**18053G**/25505 T	− 14.97 (17.53)	− 0.090	0.39	− 0.05 (0.02)	− 0.240	**0.02**
g.-82 T/**15631 T**/18053A/25505C	− 46.98 (58.90)	− 0.079	0.43	− 0.08 (0.07)	− 0.111	0.27
g.-**82C**/15631G/18053A/25505C	32.85 (56.52)	0.055	0.56	0.02 (0.07)	0.022	0.82
Sex	− 17.25 (16.04)	− 0.104	0.29	− 0.02 (0.02)	− 0.073	0.46
Activity reducing factors^a^	− 41.08 (21.40)	− 0.195	**0.05**	− 0.07 (0.02)	− 0.236	**0.02**
Activity increasing factors	65.41 (23.29)	0.308	**0.006**	0.11 (0.03)	0.395	** < 0.001**

^a^Non-genetic factors: chronic alcohol consumption, amoxicillin + clavulanic acid therapy. In haplotypes, the polymorphic variants are indicated in bold. The *P* values < 0.05 were considered to be statistically significant and are indicated in bold.

### Effect of genetic and non-genetic factors on hepatic CYP2B6 activities

**Figure 3:**
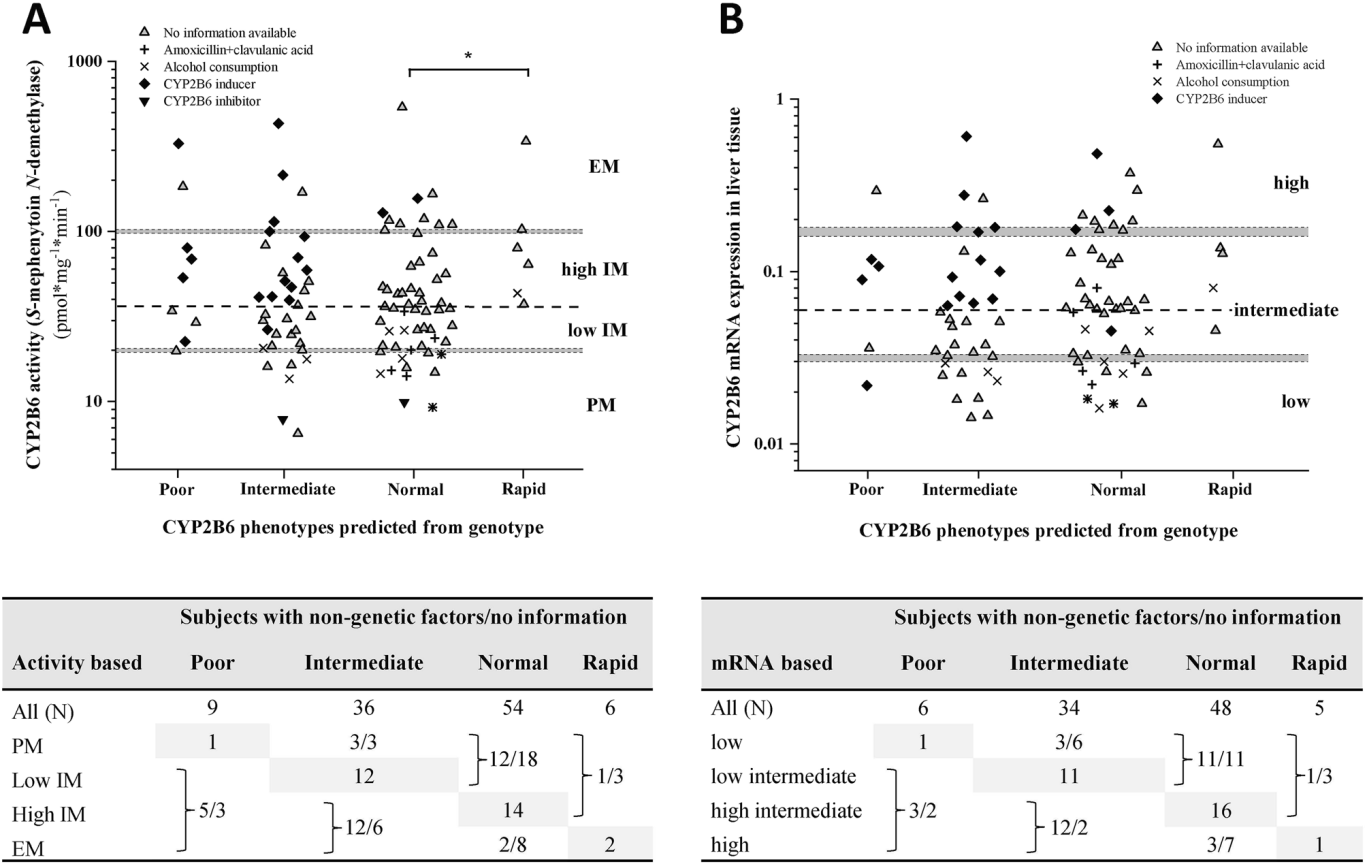
Hepatic CYP2B6 activity (*S*-mephenytoin *N*-demethylation) (**A**) and CYP2B6 expression (**B**) in subjects belonging to various *CYP2B6* genotype groups. Non-genetic factors (CYP2B6 inducer and inhibitor therapy, amoxicillin + clavulanic acid treatment, chronic alcohol consumption) found in clinical reports of the tissue donors are indicated. The median CYP2B6 activity (dotted line) is for the cutoff value between high- and low-intermediate metabolizers. Tables provide the number of subjects in each metabolizer groups with the information of relevant non-genetic factors. *PM* poor metabolizer, *IM* intermediate metabolizer, *EM* extensive metabolizer, low low expression; intermediate intermediate expression; high high expression. *CYP2B6* genotype groups: Poor CYP2B6*6/*6, CYP2B6*6/*9; Intermediate: CYP2B6*1/*6, CYP2B6*5/*6, CYP2B6*1/*9, CYP2B6*4/*6; Normal: CYP2B6*1/*1, CYP2B6*1/*5; Rapid: CYP2B6*1/*4, CYP2B6*1/*22, CYP2B6*4/*5. *P < 0.05.

For appropriate comparison, the activity-based (poor-intermediate-extensive) and genotype-based phenotype categories (poor-intermediate-normal-rapid) were harmonized. Using the median activity (36.17 pmol*mg^−1^*min^−1^) as the cut-off value, the intermediate activity category was divided into low- and high-intermediate activities corresponding to the ‘intermediate’ and ‘normal’ genotype-based phenotype categories (Table 3, Fig. 3A). Several *CYP2B6* allelic variants have been reported to significantly modify CYP2B6 activity^9^. The *CYP2B6* genotype-based phenotype prediction however seemed to be not completely consistent with the activity-based categories in liver tissue donors (N = 105). Therefore, the impact of non-genetic factors (medication, chronic alcohol consumption) on CYP2B6-selective *S*-mephenytoin *N*-demethylase activity was also investigated. The CYP2B6 inducers (dexamethasone, methylprednisolone, prednisolone, hydrocortisone, cortisone, midazolam, felodipine, diazepam) and the CYP2B6-selective inhibitor amlodipine as well as non-specific non-genetic factors (amoxicillin + clavulanic acid therapy, alcohol consumption) that can increase or decrease CYP2B6 expression and/or function were assumed to result in modification of the genetically determined CYP2B6 activity (Table 3)^25,49–57^.

Of the 9 genetically ‘poor’ metabolizer subjects with two loss-of-function alleles (CYP2B6*6/*6 or CYP2B6*6/*9 genotypes), only one displayed poor metabolism of *S*-mephenytoin, whereas the CYP2B6 activity was considered to be low/high-intermediate or even extensive in 8 tissue donors (Fig. 3A). In the medical history of these 8 donors, CYP2B6 inducer therapy (methylprednisolone, dexamethasone, diazepam) as the non-genetic factor explained the elevated activity in 5 tissue donors. According to the PharmVar, ‘intermediate’ metabolizers carrying CYP2B6*1/*6, CYP2B6*5/*6, CYP2B6*1/*9 or CYP2B6*4/*6 genotypes are expected to have low-intermediate metabolism of *S*-mephenytoin. However, CYP2B6 activities in genetically ‘intermediate’ metabolizer tissue donors (N = 36) ranged from poor (6.47 pmol*mg^-1^*min^-1^) to extensive *S*-mephenytoin *N-*demethylation (432.7 pmol*mg^−1^*min^−1^). Poor activity was attributed to CYP2B6 inhibitor (amlodipine) therapy and chronic alcohol consumption in 3 subjects, whereas CYP2B6 inducer therapy was frequently reported in the clinical history of the tissue donors with high-intermediate and extensive CYP2B6 activity (12/18 in high-intermediate/extensive metabolizers versus 1/12 in low-intermediate metabolizers, OR: 22, 95% CI: 2.27–213, *P* = 0.0024) (Fig. 3A). Most of the tissue donors carrying CYP2B6*1/*1 or CYP2B6*1/*5 genotypes (‘normal’ metabolizers, N = 54) were predicted to display high-intermediate CYP2B6 activity; however, their *S*-mephenytoin *N*-demethylase activities ranged from poor (the lowest 9.25 pmol*mg^−1^*min^−1^) to extensive metabolism (the highest 538.3 pmol*mg^−1^*min^−1^) (Fig. 3A). Despite the CYP2B6*1/*1 or CYP2B6*1/*5 genotypes, 30 tissue donors displayed lower CYP2B6 activity (poor or low-intermediate activity) than predicted from the genotype. The activity reducing non-genetic factors (CYP2B6 inhibitor amlodipine; non-specific amoxicillin + clavulanic acid therapy and chronic alcohol consumption) in the medical history was frequently indicated in the subjects with poor or low-intermediate activities (12/30 in poor/low-intermediate metabolizers versus 0/14 in high-intermediate, OR: 19.6, 95% CI: 1.07–359.6, P = 0.0084). Of the 10 extensive metabolizers, 2 were on CYP2B6 inducer therapy (methylprednisolone, midazolam) that confirmed the high CYP2B6 activity. ‘Rapid’ metabolizer phenotype predicted from the genotype (CYP2B6*1/*4, CYP2B6*1/*22 or CYP2B6*4/*5) was identified in 6 tissue donors; however, only 2 displayed extensive *S*-mephenytoin *N*-demethylation, and in the medical history of one subject, chronic alcohol consumption appeared to be responsible for high-intermediate metabolism. In conclusion, *CYP2B6* genetic polymorphisms explained *S*-mephenytoin *N*-demethylase activity in not more than 29 tissue donors (27.6%), and considering non-genetic factors improved the phenotype prediction by further 33.3% (35 tissue donors); however, in the medical history of 41 subjects, no relevant information on the non-genetic factors for altered CYP2B6 metabolism was found. It also means that phenoconverting non-genetic factors in these 41 subjects could not confirm altered CYP2B6 phenotype predicted from genotype, and genotype–phenotype mismatch still existed in 39.1% (41/105) of the subjects.

### Effect of genetic and non-genetic factors on hepatic CYP2B6 mRNA expression

The nucleotide change g.-82 T > C (rs34223104) has been reported to be associated with increased expression of CYP2B6 mRNA, whereas g.15631G > T (rs3745274) is associated with an aberrant mRNA splicing variant lacking exons 4 to 6 and entailing reduced CYP2B6 function or reduced expression of the full-length CYP2B6 mRNA variant^11,19^. In the present study (N = 93), we evaluated the association of hepatic CYP2B6 expression with *CYP2B6* genotypes as well as with non-genetic phenoconverting factors, such as CYP2B6 inducer therapy (diazepam, dexamethasone, methylprednisolone, felodipine, cortisone, midazolam), non-specific amoxicillin + clavulanic acid therapy and chronic alcohol consumption (Fig. 3B).

Low CYP2B6 mRNA expression was expected in those individuals (N = 6) carrying two loss-of-function alleles (CYP2B6*6/*6 and CYP2B6*6/*9 genotypes) because of the truncated CYP2B6 splicing variant. However, there was more than tenfold difference between the lowest (0.02179) and highest (0.2932) mRNA expression. In the medical history of 3 subjects with high-intermediate expression and surprisingly of one with low expression, CYP2B6 inducer therapy (4/6; diazepam, dexamethasone, methylprednisolone) was indicated. The ‘intermediate’ genotype-based phenotype category was applied for those subjects with one normal function and one loss-of-function alleles (CYP2B6*1/*6, CYP2B6*5/*6, CYP2B6*1/*9, CYP2B6*4/*6 genotypes), and was predicted to display low-intermediate CYP2B6 mRNA expression (N = 34). In fact, we found high variability (42-fold) between the lowest and the highest mRNA expressions (0.01418 and 0.60709). The low CYP2B6 expression in 3 liver tissue samples was explained by chronic alcohol consumption (3/9 in low expressers vs. 0/11 in low-intermediate expressers, OR: 12.39; 95% CI: 0.549–279.4; *P* = 0.073). Treatment with CYP2B6 specific inducers (felodipine, cortisone, methylprednisolone) was recorded in the majority of tissue donors with high or high-intermediate mRNA expression (12/14 in high/high-intermediate vs. 0/11 in low-intermediate; OR: 115; 95% CI: 4.973–2659.5; *P* < 0.0001). The subjects with two normal function alleles (CYP2B6*1/*1 and CYP2B6*1/*5 genotypes) were estimated to have high-intermediate mRNA expression (N = 48). However, we found several tissue donors with CYP2B6 expression different from genotype-based prediction, with low and low-intermediate as well as with high CYP2B6 mRNA levels (22/48 and 10/48). Phenoconverting effect of amoxicillin + clavulanic acid therapy and/or chronic alcohol consumption explained low and low-intermediate mRNA expression in half of the liver tissues (11/22 in low and low-intermediate versus 1/16 in high-intermediate; OR: 15; 95% CI: 1.678–134.1; *P* = 0.0051). In the medical history of 3 tissue donors with high mRNA expression (3/10), CYP2B6 inducer therapy (methylprednisolone, midazolam, dexamethasone) was recorded (3/10 high expressers versus 0/16 in high-intermediate; OR: 15.4, 95% CI: 0.703–337.5, *P* = 0.0462). Although the individuals with one normal and one gain-of-function alleles (N = 5; CYP2B6*1/*4 and CYP2B6*1/*22 genotypes) were predicted to display ‘rapid’ metabolizer phenotype, CYP2B6*22 carriers were expected to have high CYP2B6 mRNA expression (2/5). Four tissue donors (2 with CYP2B6*1/*4 and 2 with CYP2B6*1/*22) expressed CYP2B6 at low-intermediate and high-intermediate levels, and only one subject with CYP2B6*1/*4 genotype was high CYP2B6 expresser (1/5). Chronic alcohol consumption was recorded for one tissue donor with CYP2B6*1/*4 genotype. In conclusion, CYP2B6 mRNA expression of less than one third of the liver tissue samples (29 tissue donors, 31.2%) was confirmed by *CYP2B6* genotype, and non-genetic factors recorded in the medical history of the donors explained altered CYP2B6 expression in further 33 liver samples (35.5%). For the remaining 31 tissue donors (33.3%), no relevant information explained CYP2B6 mRNA expression different from the genotype-based phenotype prediction.

### Multivariate analysis of CYP2B6 activity and mRNA expression

Multiple linear regression analysis was performed to estimate the influence of genetic (*CYP2B6* SNVs or haplotypes) and non-genetic covariates (sex, medication with CYP2B6 inducers or amoxicillin + clavulanic acid, chronic alcohol consumption) on CYP2B6 activity and on CYP2B6 mRNA expression (Table 4). Significant associations were observed between *S*-mephenytoin *N*-demethylation activity and the nucleotide substitution g.15631G > T (*P* = 0.034) or the CYP2B6 inducer therapy (*P* = 0.005). When the *CYP2B6* haplotypes were involved in the analysis, the impact of none of the haplotypes containing g.15631 T was significant (g.-82 T/15631 T/18053G/25505 T *P* = 0.383; g.-82 T/15631 T/18053A/25505 T *P* = 0.427), whereas CYP2B6 activity-reducing non-genetic factors (chronic alcohol consumption and amoxicillin + clavulanic acid therapy) appeared to be associated with CYP2B6 activity (*P* = 0.050). Furthermore, hepatic CYP2B6 mRNA expression was found to be significantly associated with the nucleotide substitution g.15631G > T (*P* = 0.004) and also with g.18053A > G (*P* = 0.038). Involving *CYP2B6* haplotypes in the multiple regression model, the association between CYP2B6 mRNA expression and the g.-82 T/15631 T/18053G/25505 T haplotype present in CYP2B6*6 allele became significant (*P* = 0.025). Both the CYP2B6 inducer therapy and the expression reducing non-genetic factors displayed significant association with hepatic CYP2B6 expression (*P* < 0.001 and *P* = 0.021, respectively). However, sex appeared to have no influence on either CYP2B6 activity or mRNA expression.

## Discussion

Genetic variability of *CYP2B6* has been reported to be associated with significant interindividual variations in pharmacokinetics of several clinically important drugs (antiretroviral, anticancer, antidepressant, antimalarial drugs)^58^. Moreover, preliminary pharmacogenetic testing is highly recommended for patients on efavirenz therapy for proper therapeutic efficacy and for limitation of adverse reactions^23^. Identification of SNVs in *CYP2B6* gene and haplotype estimation constitute a major challenge, because for *CYP2B6* genotyping, reliable and *CYP2B6*-selective assays are required that can distinguish *CYP2B6* sequences from the highly homologous pseudogene *CYP2B7P*. TaqMan PCR assays offer accurate, sensitive, cost-efficient and fast SNV-discrimination method. Validated TaqMan *CYP2B6* genotyping assays are commercially available for g.-82 T > C, g.15631G > T and g.25505C > T polymorphisms, but not for g.18053A > G, which is present in many allelic variants including CYP2B6*4 and the most frequent and widely studied CYP2B6*6; therefore, for identification of g.18053A > G, we have developed a novel, two-step genotyping assay. In the 119 liver samples, the frequencies of *CYP2B6* alleles and genotypes in liver tissue donors were demonstrated to be similar to those in Caucasian populations (Table 2) (https://www.pharmgkb.org/page/cyp2b6RefMaterials, access date: 24.01.2022)^8^. Although genetic polymorphisms of *CYP2B6* can elucidate the substantial interindividual variability in CYP2B6 expression and activity to some extent, non-genetic factors can significantly modify the CYP2B6 phenotype predicted from genotype. The present study investigated the contribution of *CYP2B6* genetic and non-genetic factors to CYP2B6-selective *S*-mephenytoin *N-*demethylation and CYP2B6 mRNA expression as well as the *CYP2B6* genotype–phenotype mismatch in human liver tissues. *S*-Mephenytoin as the probe substrate and its *N*-demethylation reaction is frequently used for characterization of hepatic microsomal CYP2B6 activity^59^; however, only a few studies have applied this CYP2B6-selective reaction in genotype–phenotype analysis^21,30,60^. In CYP2B6 expression analysis, the primer pair was designed to the exons 3 and 4 for the quantification of the full-length CYP2B6 mRNA. The strong association between CYP2B6 mRNA and *S*-mephenytoin *N*-demethylase activity in human liver tissues proved that the amplicon produced in the quantitative PCR was appropriate for the identification of the functional CYP2B6 mRNA and did not detect the truncated mRNA variant.

Several in vitro and in vivo studies indicated that some *CYP2B6* allelic variants may have substrate-specific effect on CYP2B6 function that further complicates the CYP2B6 phenotype estimation based on PharmVar criteria system^8,9^. PharmVar classification is appropriate for *CYP2B6* genotype guided efavirenz therapy, whereas *CYP2B6* pharmacogenetics appear to have an opposite impact on cyclophosphamide bioactivation. The most common CYP2B6*6 is associated with the expression of an mRNA variant lacking exons 4–6 due to aberrant splicing, and consequently with decreased hepatic activity in efavirenz and bupropion metabolism^18,19,22^. Efavirenz exposure has been reported to increase in patients with CYP2B6*1/*6 or CYP2B6*6/*6; therefore, substantial dose reduction has been recommended for better tolerability^15,23^. Although the presence of CYP2B6*6 allele appeared to have minor or negligible effect on bupropion exposure, hydroxylation of both enantiomers was lower in patients carrying CYP2B6*6/*6^17,61^. Contradictory results have been reported on metabolic activation of the prodrug cyclophosphamide in hepatic microsomes from CYP2B6*6 carriers^18,62,63^; however, lower 4-hydroxy-cyclophosphamide production and worse treatment response to cyclophosphamide was observed in patients with CYP2B6*6 allele than in CYP2B6*6 non-carriers^64^. The g.18053A > G nucleotide change in CYP2B6*4 allelic variant has been reported to significantly alter the enzyme structure leading to a functionally different protein variant and to increased drug-metabolizing activity^12,13,15–17,65^. The CYP2B6*4 allele contributed to increased activity in efavirenz 8-hydroxylation and to reduced plasma concentration of efavirenz in HIV-infected patients^15,18^; however, dose modification was not required for efficient efavirenz therapy^23^. Intrinsic clearance of both bupropion enantiomers was minimally increased by CYP2B6*4, whereas pharmacokinetic studies demonstrated significantly high bupropion clearance in vivo and consequently high hydroxy-bupropion exposure in CYP2B6*4 carrier subjects^17,61,66^. Interestingly, CYP2B6*4 displayed lower cyclophosphamide 4-hydroxylation activity in vitro than CYP2B6*1^18,63^; however, CYP2B6*1/*4 genotype appeared to have no impact on 4-hydroxy-cyclophosphamide formation in vivo comparing to CYP2B6*1/*1^64^. In the liver tissues of the present study, CYP2B6 mRNA expression was significantly associated with the g.15631C > T and g.18053A > G SNVs and even more with the g.-82 T/15631 T/18053G/25505 T haplotype designated as CYP2B6*6. *S*-Mephenytoin *N*-demethylase activity appeared to be significantly influenced by g.15631C > T, and marginally significant impact of g.18053A > G was demonstrated; however, the haplotype g.-82 T/15631 T/18053G/25505 T (CYP2B6*6) did not affect CYP2B6 activity at all.

Besides *CYP2B6* genetic variations, phenoconverting non-genetic factors, such as sex, age, co-medication and co-morbidities, have been considered to contribute to the interindividual variability in CYP2B6 activity and expression^9,29,58^. Al Koudsi et al. attributed 10% of variations in CYP2B6 protein expression to *CYP2B6* genotype, 14% to gender and 21% to exposure to hepatic CYP inducers^32^. Several studies indicated that females displayed significantly higher CYP2B6 expression and activity than males^30,32,67^. It was explained by estradiol induction through an estrogen response element in the regulatory region of *CYP2B6* gene^68,69^. However, other studies, including the present work, demonstrated no association between gender and CYP2B6 phenotypes^19,31,33,34^. Environmental non-genetic factors, such as CYP2B6-specific or non-specific medication and the consequences of chronic alcohol consumption are expected to contribute to the high interindividual variability in CYP2B6 expression and activity. Of less than 30% of liver tissue donors, *CYP2B6* genetic variability influenced the CYP2B6 phenotype, whereas of more than 35% of tissue donors, non-genetic factors were reported in the medical history that significantly altered *S*-mephenytoin *N*-demethylase activity and/or CYP2B6 mRNA expression. In addition to genetic variations, the impact of both CYP2B6-selective inhibitors and inducers is highly recommended to be taken into account during CYP2B6 phenotype prediction^8,9,29^. The function of several CYP enzymes have been demonstrated to be inhibited by 1,4-dihydropyridine calcium-channel antagonists, including amlodipine that was found to strongly inhibit in vitro activities of CYP2B6 and CYP3A4; however, the clinical significance of the interaction between amlodipine and CYP2B6 might be minor because of the relatively high IC_50_ values towards CYP2B6 substrates^49,70^. In two subjects, one with CYP2B6*1/*1, the other with CYP2B6*5/*6 genotype, and predicted to be ‘normal’ and ‘intermediate’ metabolizers, respectively, the low *S-*mephenytoin *N*-demethylation was attributed to the antihypertensive amlodipine therapy that might have transiently evoked poor CYP2B6 activity. Furthermore, the exposure to CYP2B6 inducers, including the antibiotics rifampicin, the corticosteroid derivative prednisolone, cortisone, hydrocortisone and dexamethasone, the benzodiazepine diazepam and midazolam, and the calcium channel blocker felodipine induces transcriptional expression of *CYP2B6* gene via nuclear receptors (PXR, CAR)^25,50,52,53,55,57,71^. Rifampicin treatment has been demonstrated to substantially increase the clearance of bupropion and the formation of hydroxy-bupropion metabolite^72^. Furthermore, selective activation of CAR has been found to lead to increased bioactivation of cyclophosphamide in hepatocytes and to enhanced cytotoxicity in leukemia cells^73^. Metabolic activation of cyclophosphamide is primarily catalyzed by CYP2B6 with minor contribution of CYP3A4, whereas CYP3A4 is responsible for the inactivation pathway^74^. Since CAR preferentially mediates transcriptional induction of CYP2B6 over CYP3A4, CAR activation resulted in an increase in the active metabolite formation and in elevated antitumor activity^73^. In the liver tissue donors, the CYP2B6 inducer therapy in their medical history (dexamethasone, methylprednisolone, prednisolone, hydrocortisone, cortisone, midazolam, felodipine, diazepam) was significantly associated with increased CYP2B6 mRNA expression and activity. Particularly in those with one or two loss-of-function alleles predicted to be ‘intermediate’ or ‘poor’ metabolizers, high or high-intermediate activity and expression were observed. The loss-of-function alleles (CYP2B6*6 and CYP2B6*9) display some residual expression and activity; therefore, it was logically assumed that the exposure to a CYP2B6 inducer ameliorated the reduced function of CYP2B6 predicted from genotype. The clinical study by Loboz et al. involving healthy volunteers demonstrated that rifampicin induction increased bupropion clearance even in those carrying the loss-of-function CYP2B6*6 allele^72^. As a consequence of rifampicin treatment (PXR activation), hydroxy-bupropion formation was enhanced in subjects with CYP2B6*1/*6 as well as in CYP2B6*1/*1 carriers^72^. In the liver tissue donors of the present study, increased *S*-mephenytoin *N*-demethylation activity and CYP2B6 mRNA expression were associated with CYP2B6 inducer therapy, and 83% of the subjects exposed to CYP2B6 inducers (19/23) carried one or two copies of CYP2B6*6 allele; however, for the clear evidence for the CYP2B6*6-dependent susceptibility to induction, further study involving a large population is required. The polymorphism of g.-82 T > C has nevertheless been demonstrated to be associated with *CYP2B6* genotype-dependent susceptibility to rifampicin induction due to increased recruitment of PXR to the promoter region in g.-82C carriers^10^. Genotype-dependent susceptibility to CYP2B6 inhibitors has also been reported by Talakad et al. demonstrating an increase in inhibitory constant (K_i_) values of the CYP2B6*4 and CYP2B6*6 variants with sertraline or clopidogrel compared to the wild-type enzyme^75^. However, CYP2B6*6 was found to be more susceptible to voriconazole inhibition than CYP2B6*1^76^, indicating inhibitor-dependent susceptibility of CYP2B6*6. Duration of phenoconversion and the recovery after CYP2B6 inhibition or induction have been reported to depend on the elimination rate of the inhibitor or inducer drugs and/or on enzyme turnover^77^. In the liver tissue donors, the putative time-course of drug-induced phenoconversion might have influenced the hepatic CYP2B6 activity and expression after discontinuation of the CYP2B6 inhibitor amlodipine or CYP2B6 inducer drugs. Considering the fact that the information on the drug therapy (both chronic and acute) applied prior the brain-death was recorded in the clinical histories, and the time of tissue explantation never exceeded 3 h, we assumed that no loss of altered enzyme activity or expression occurred in liver samples. Amlodipine and the CYP2B6 inducers are drugs with relatively long half-lives (12–60 h); furthermore, the de-induction and the recovery of CYP enzyme after inducer discontinuation has been calculated to require 3–7 days^78,79^; therefore, we considered that phenoconversion evoked by these drugs still existed at the time of explantation.

Furthermore, reduced CYP2B6 activity and/or expression in liver tissues was also associated with the non-specific amoxicillin + clavulanic acid therapy and/or chronic alcohol consumption. Amoxicillin, effective against a wide range of bacterial infections, is often used in combination with clavulanic acid that prevents bacterial metabolism of amoxicillin^80^. Moderately severe hepatotoxic side effect of this combination has been reported; severe hepatic dysfunction however rarely occurs^56,81^. Chronic alcohol consumption is one of the major causes of liver diseases, the progression of which is explained by several pathological processes (e.g. inflammation, oxidative stress); however, the exact pathomechanism is not clearly understood^54^. No information is available about CYP2B6-selective inhibition or downregulation induced by amoxicillin + clavulanic acid or chronic alcohol consumption; however, hepatotoxicity and the associated inflammatory processes were assumed to influence CYP expression and function^82^. Clear evidence has been provided that the release of proinflammatory cytokines during inflammation downregulates both protein or mRNA expression of several CYPs, including CYP2B6^83,84^. IL-6 (interleukin 6) and IFNγ (interferon γ) proinflammatory cytokines have been demonstrated to downregulate the expression of CYP2B6 mRNA and protein as well as CYP2B6 activity^51,85–88^. In the liver tissue donors with amoxicillin + clavulanic acid therapy and/or chronic alcohol consumption, decreased CYP2B6 expression and *S*-mephenytoin *N*-demethylase activity were attributed to a non-specific impact of inflammatory processes on CYP function rather than to a CYP2B6-selective suppression.

The present work indicated that *CYP2B6* genetic polymorphisms influenced the expression and activity of CYP2B6 enzyme to some extent; however, the significance of phenoconverting non-genetic factors in enzyme function was comparable with that of genetic factors or even phenoconversion masked the effect of *CYP2B6* allelic variants. Ing Lorenzini et al. evaluated the predictive values of CYP genotypes on CYP-mediated drug metabolism in patients, and observed relatively good CYP2B6 genotype–phenotype concordance for poor and rapid metabolizers (67% and 100%), but more variable for intermediate and normal metabolizers (0% and 38%)^89^. It should be noted that the number of patients involved in the interpretation of CYP2B6 genotype–phenotype concordance was limited (N = 36). In a retrospective study involving patients taking analgesic drugs for chronic pain, genetic variability and non-genetic factors influencing drug-metabolizing enzyme activities were associated with the occurrence of adverse drug reactions and/or non-response to the therapy in 40% and 28% of the cases^90^.

Some limitations of the present work should be discussed. First, we assessed the impact of *CYP2B6* alleles most common in Caucasian populations, and some other, functionally relevant allelic variants were not identified (CYP2B6*7 or CYP2B6*12); however, their prevalence is low in Caucasian populations. Second, the medical history of some tissue donors may be assumed to be incomplete, and some information about relevant non-genetic factors was missing that might have influenced the interpretation of CYP2B6 phenotypes. Certain co-morbidities have been demonstrated to impact the function of drug-metabolizing enzymes; however, in the clinical histories of the tissue donors, the information of relevant pathological conditions was scarcely or incompletely recorded.

Although pharmacogenetic testing of drug-metabolizing CYP enzymes is an effective approach towards optimization and personalization of drug therapy^23,91^, the assessment of patients’ drug-metabolizing capacity is far more complex than a simple prediction from the genotype. The present work has demonstrated that both CYP2B6 genetic and non-genetic variations were important to be taken into account in CYP2B6 phenotype interpretation. However, in approximately one third of the subjects, a CYP2B6 genotype–phenotype mismatch still existed. Identifying potential factors (both CYP2B6-specific and non-specific factors) in CYP2B6 phenotype variability and considering both genetic variations and non-genetic factors is a pressing requirement for appropriate elucidation of CYP2B6 genotype–phenotype mismatch that may improve prediction of pharmacokinetic variations and clinical outcome of a drug that is primarily or exclusively metabolized by CYP2B6 enzyme (e.g. cyclophosphamide, efavirenz, bupropion, ketamine, methadone).

## Supplementary Information

Supplementary Information.

## Abbreviations

CAR: Constitutive androstane receptor
CPIC: Clinical pharmacogenetics implementation consortium
Ct: Threshold cycle
CYP: Cytochrome P450
GAPDH: Glyceraldehyde 3-phosphate dehydrogenase
PCR: Polymerase chain reaction
PharmVar: Pharmacogene Variation Consortium
PXR: Pregnane X receptor
SNV: Single nucleotide variation
