# Supplementary material for: CYP2B6 allelic variants and non-genetic factors influence CYP2B6 enzyme function
Source: Sci Rep. 2022 Feb 22;12:2984. doi: 10.1038/s41598-022-07022-9 (PMC8863776; doi:10.1038/s41598-022-07022-9)
Supplement: Supplementary file 1 — Supplementary Information. [file 41598_2022_7022_MOESM1_ESM.pdf]

Supplementary Table 1. Demographic data of liver tissue donors

**Demographic data**			N
**Donor number**			119
**Age (year)** [a]			47 (18;74)
**Gender**	Female/male		52/67
**Cause of death**	Cerebral hemorrhage/hematoma	Subarachnoid hemorrhage	25
		Subdural hemorrhage	6
		Intraventricular hemorrhage	7
		Ruptured cerebral aneurysm	5
		Unknown	4
	Stroke	Ischemic stroke	8
		Hemorrhagic stroke	2
	Tumour		34
	Accident	Car/motor/bike accident	13
		Seizure induced cerebral injury	1
		Suicide	1
		Unknown cerebral injury	5
	Unknown		8
**Medical history**	Amoxicillin+clavulanic acid therapy		7
	Chronic alcohol consumption		11
	CYP2B6 inducer therapy		23
	CYP2B6 inhibitor therapy		2

[a] median (min; max)

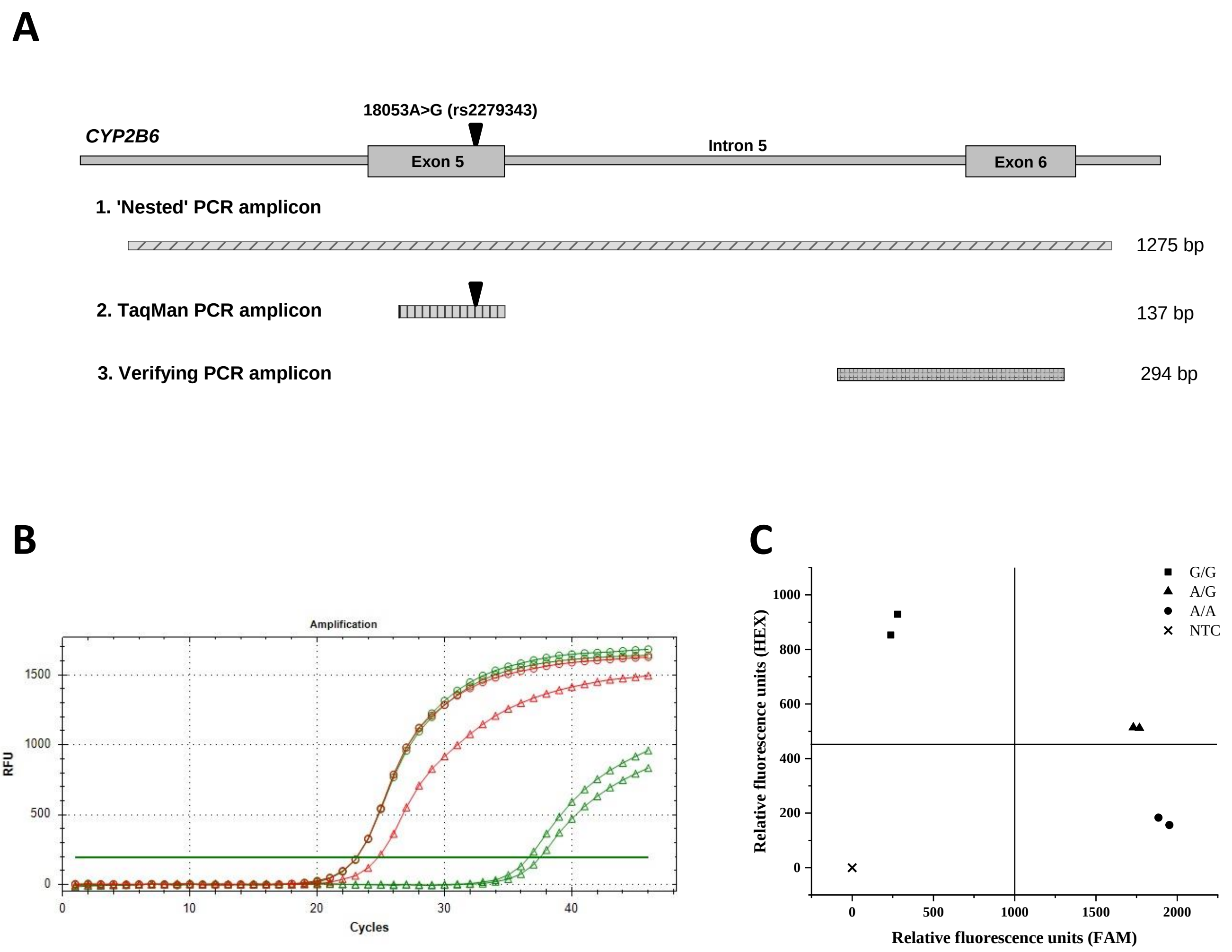

Supplementary Figure 1. Identification of g.18053A>G in *CYP2B6* (rs2279343). A) Proportionate illustration of the PCR amplicons of the two-step PCR assay based on the ‘nested’ PCR method with ‘touchdown’ thermal cycling protocol and the TaqMan PCR, also the verification PCR. B) Verifying *CYP2B6* specificity of the pre-amplification step of ‚nested’ PCR. Substantial amount of *CYP2B6* specific amplicon (amplification curves marked with „circles”) were produced over *CYP2B7P* specific amplicon (amplification curves marked with „triangles”) using pre-amplified samples (green amplification curves) and genomic DNA samples (red amplification curves). C) Allele-discrimination of g.18053A>G (rs2279343) genotyping reaction.

Homozygous wild type (g.18053A/A)

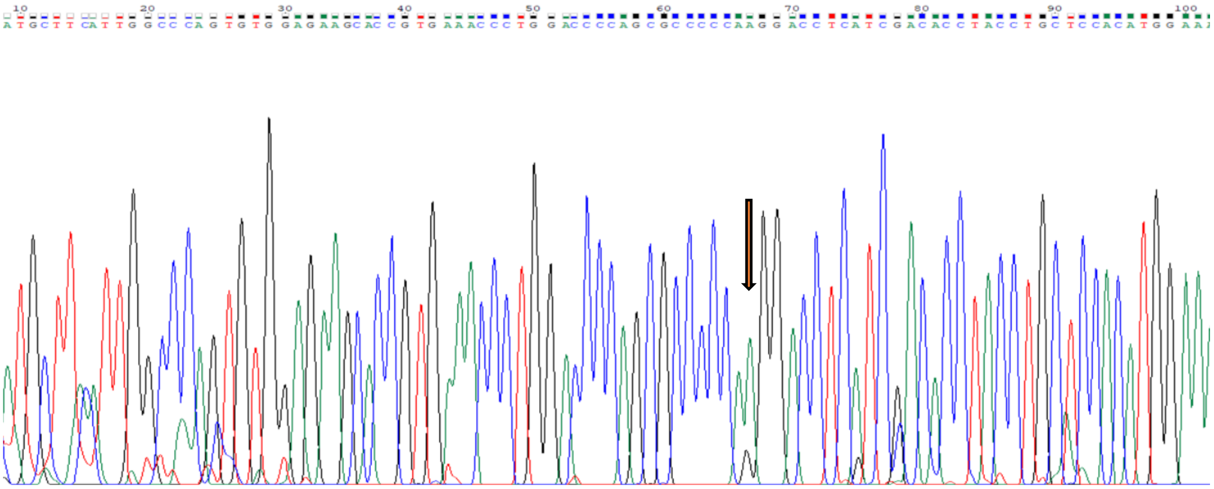

Heterozygous (g.18053A/G)

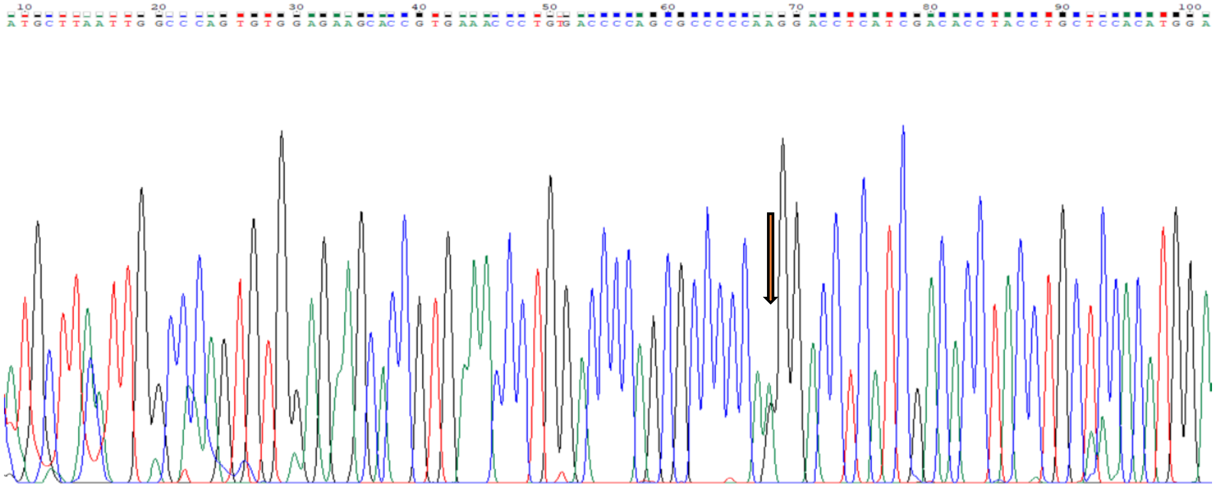

Homozygous mutant (g.18053G/G)

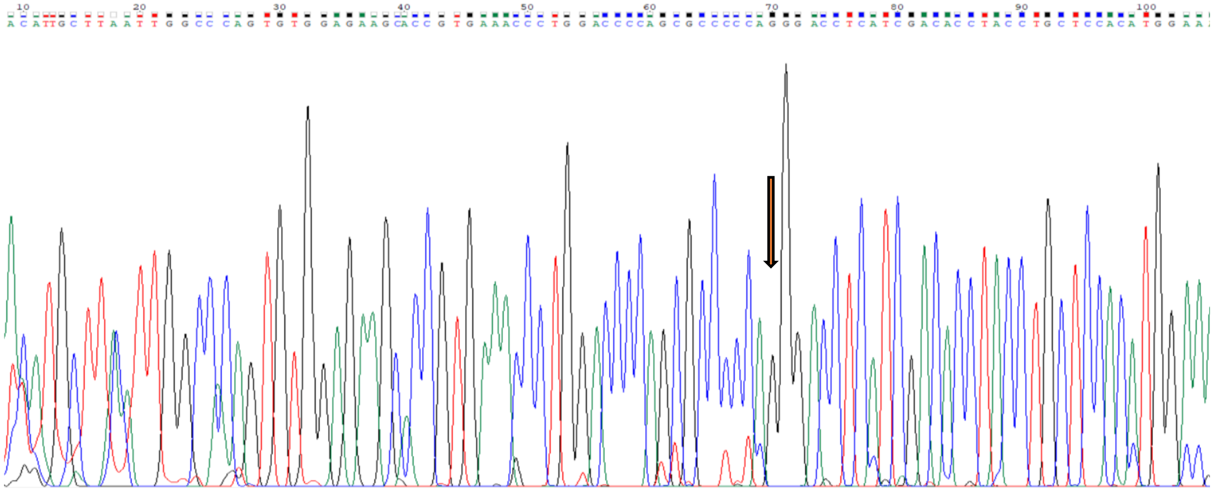

Supplementary Figure 2. The accuracy of g.18053A>G (rs2279343) SNV-discrimination was proven by Sanger-sequencing of a homozygous wild (g.18053A/A), a heterozygous (g.18053A/G) and homozygous mutant (g.18053G/G) type samples.
